# Advancing Geriatric Care: The Impact and Future of STRIDE in the VA

**DOI:** 10.1093/geroni/igaf122.484

**Published:** 2025-12-31

**Authors:** Caitlin Kappler, Karen Stechuchak, Amy Webster, Ashley Choate, Susan Hastings

**Affiliations:** Durham Veterans Affairs, Durham, North Carolina, United States; Durham VA Health Care System, Durham, North Carolina, United States; Durham VA Health Care System, Durham, North Carolina, United States; Durham VA Health Care System, Durham, North Carolina, United States; Durham VA Healthcare System, Durham, North Carolina, United States

## Abstract

STRIDE (AssiSTed EaRly MobIlity for HospitalizeD VEterans) is an evidence-based, supervised walking program designed to preserve and enhance the physical function of older Veterans, a population at high risk for hospital-associated disability. As the VA serves an aging Veteran community, ensuring access to effective geriatric care is critical for maintaining independence and quality of life. Launched in 2014, STRIDE has been successfully implemented in over 70 Veterans Affairs (VA) hospitals nationwide, proactively addressing functional decline through early intervention. The program encourages enrollment ideally within 24 hours of admission, provides supervised daily walks of up to 20 minutes, and ensures dedicated staff oversee both the walks and pre/post evaluations. Evidence demonstrates that STRIDE participants experience shorter hospital stays, reduced discharge to skilled nursing facilities, and report feeling better immediately after walking. A robust set of standardized implementation support tools has been shown to facilitate STRIDE’s adoption in VA hospitals, providing a scalable solution to mitigate hospital-associated disability. Sustaining and expanding STRIDE requires ongoing investment in research and implementation support, particularly in securing dedicated staffing and fostering leadership engagement at local, regional, and national levels. By improving outcomes for aging Veterans, STRIDE strengthens the VA’s capacity to integrate and sustain best practices, ensuring older Veterans receive the specialized care they need to maintain mobility, independence, and overall well-being.

